# Research intensity for neglected tropical diseases and other infectious diseases before and after the COVID-19 pandemic

**DOI:** 10.7189/jogh.16.04255

**Published:** 2026-08-07

**Authors:** Ryo Komorizono, Raita Tamaki, Naoko Fujita, Masakaze Hamada, Kana Suzuki, Satoshi Kaneko, Yuki Furuse

**Affiliations:** 1 Department of Microbiology and Infection, The University of Tokyo Pandemic Preparedness, Infection and Advanced Research Centre (UTOPIA), The University of Tokyo, Tokyo, Japan; 2 Kenya Research Station, Institute of Tropical Medicine, Nagasaki University, Nagasaki, Japan; 3 Department of Eco-epidemiology, Institute of Tropical Medicine, Nagasaki University, Nagasaki, Japan

**Keywords:** neglected tropical diseases, COVID-19, research and development, bibliometrics, disease burden, equity

## Abstract

**Background:**

Infectious diseases can cause tremendous health and economic damage, as shown by past pandemics. Although research tends to spotlight emerging infectious diseases, neglected tropical diseases (NTDs) require sustained research and development to advance global public health. However, there is concern that the recent COVID-19 pandemic may have interrupted research on NTDs, leading to further neglect of these diseases.

**Results:**

We assessed the bias of research intensities for 67 infectious diseases, including 18 NTDs, from 2016 to 2023. We calculated the burden-adjusted research intensity (BARI) index for each disease, based on disease burden and scholarly publications. Significantly high research intensity was observed for COVID-19, HIV, influenza, and Zika, while paratyphoid fever showed significantly low BARI. We found no significant difference in the median BARI index between NTDs and non-NTDs during the pandemic period of 2020–2023. Among the lowest quartile of BARI index scores, seven were still NTDs. The BARI index for cysticercosis, dengue, and schistosomiasis has declined significantly when comparing the pre- and post-pandemic periods. As for non-NTDs, the BARI of 12 diseases decreased; there was no significant difference in the proportion of diseases for which the BARI decreased during the COVID-19 pandemic between NTDs and non-NTDs. Disease characteristics and research locations were modestly associated with research intensity, and the impact of COVID-19 was not evident even in the subgroups.

**Conclusions:**

We found no evidence that NTDs, as a formal disease category, were disproportionately deprioritised in research during the COVID-19 pandemic. However, several diseases, including both NTDs and non-NTDs, showed persistently low or declining relative research intensity. As research on non-pandemic infectious diseases, including NTDs, should be viewed not as competing with pandemic preparedness and response, but as integral to it, this study emphasises the importance of sustained, wide-reaching research investments for global equity.

Infectious diseases continue to be a significant threat to global populations, claiming approximately 15 million lives annually [1]. Beyond their public health impact, infectious diseases impose severe constraints on economic development, education, and social stability, as clearly demonstrated by the COVID-19 pandemic [2–4]. The COVID-19 pandemic triggered an unprecedented concentration of global research effort. Within an extremely short time frame, vast financial, human, and infrastructural resources were mobilised toward COVID-19 research [5–7], resulting in rapid advances in diagnostics, surveillance, epidemiological investigation, and the development of effective medical countermeasures including vaccines and antivirals. These achievements demonstrated the remarkable capacity of the global scientific community to rapidly respond to an infectious disease crisis. At the same time, however, the scale and speed of this pivot raised serious concerns about unintended consequences for research on other infectious diseases, particularly those already suffering from chronic underinvestment.

Neglected tropical diseases (NTDs) predominantly affect populations in low- and middle-income countries and are closely linked to poverty, inadequate sanitation, limited access to healthcare, and local ecological and climate factors. Despite causing substantial morbidity and long-term socioeconomic harm, NTDs have historically attracted disproportionately little research investment, given their disease burden [8,9]. The geographical concentration of NTDs in the Global South often leads to their exclusion from global research priorities, underscoring the need for sustained international cooperation and support [10]. To achieve effective control and ultimately eradicate NTDs, research activities must extend beyond endemic regions and be integrated into broader global health strategies [11].

Moreover, NTDs are heterogeneous in their characteristics. Some NTDs are further restricted to specific regions within the Global South, such as African trypanosomiasis in Africa and Chagas disease in South America, whereas others have broader geographic distributions, such as rabies and trachoma. Similarly, some cause chronic illnesses (*e.g.* leprosy), while others cause acute infections (*e.g.* dengue). Transmission routes and the availability of medical countermeasures for treatment and prevention also differ across NTDs. This heterogeneity should also be considered when evaluating research prioritisation.

To combat infectious diseases, limited resources must be effectively mobilised and distributed for global health. While emergent outbreaks naturally attract significant research attention [7], chronic and endemic infectious diseases, such as NTDs, should not be disproportionately neglected. In our previous study, we introduced the burden-adjusted research intensity (BARI) index to quantify mismatches between disease burden and research activities [12]. That study demonstrated that several NTDs had been persistently underrepresented in research activity relative to their burden from 1990 to 2017, well before the emergence of COVID-19.

Against this background, the COVID-19 pandemic posed a critical question for global health research equity: did the extraordinary surge in pandemic-related research further deprioritise NTDs, or did research on these diseases benefit from the global attention to infectious diseases? Many researchers were compelled to suspend their prior work and shift their focus to COVID-19 research [6], potentially reshaping the landscape of infectious disease research [5,13]. In fact, the percentage of internationally coauthored publications decreased during the pandemic [14]. We here extend our earlier BARI-based analysis into and beyond the COVID-19 pandemic period, incorporating data through 2023.

## METHODS

### Data

We analysed 67 infectious diseases, including 18 NTDs (Table S1 in the **Online Supplementary Document**). We selected 67 diseases based on the availability of disease burden data in the Global Burden of Disease (GBD) study [15]. NTDs were identified from the list published by the World Health Organization (WHO) and the United States Centers for Disease Control and Prevention [16,17]. Among them, NTDs caused by non-infectious entities (*e.g.* snakebite envenoming), those without a single identifiable causative agent (*e.g.* noma), and diseases for which reliable estimates of the global disease burden are unavailable (*e.g.* yaws and Buruli ulcer) were excluded from this study (Table S2 in the **Online Supplementary Document**).

We downloaded data on disability-adjusted life years (DALYs) from 2016 to 2023 using the GBD results tool on the Institute for Health Metrics and Evaluation website. In the database, DALYs were tabulated by the aetiology of infectious diseases and by the cause of death or injury. As for aetiology classification, 46 pathogens were listed. From them, categories that do not specify a microbial species name (*e.g.* ‘other gram-negative bacteria’) were excluded from our analysis. As for the cause of death or injury, 381 items were listed at the subcategory level. Of them, 85 were infectious diseases with identifiable aetiology. For example, COVID-19, as a cause of death or injury, was included in the present study, whereas ‘otitis media’ was not. DALYs from subcategories sharing the same aetiology were integrated into a single category: ‘drug-susceptible tuberculosis,’ ‘Multidrug-resistant tuberculosis without extensive drug resistance,’ ‘extensively drug-resistant tuberculosis,’ and ‘latent tuberculosis infection,’ all of which were listed in the subcategory of the cause of death or injury, were unified as a single disease. In this case, there was no category for *Mycobacterium tuberculosis* in the aetiology list in the GBD database. After the curation process described above, we combined the ‘aetiology’ and ‘cause of death or injury’ lists to create our final data set, comprising 67 infectious diseases (Table S1 in the **Online Supplementary Document**).

The number of publications for each disease in the corresponding period was obtained from PubMed, which is based on the MEDLINE database, using the ‘rentrez’ package in *R*, version 4.3.2 (R Core Team, Vienna, Austria). Disease-specific publication searches were conducted using MeSH terms to minimise ambiguity arising from overlapping nomenclature for etiological agents and diseases, and to reduce the risk of missing relevant records due to spelling or terminology variations. MeSH is a controlled vocabulary (*i.e.* a standardised set of terms) to index and organise biomedical literature in MEDLINE. All articles in the database are tagged with relevant MeSH terms, which represent the main topics of the papers. Data were retrieved between February 2025 and April 2026 (Table S1 in the **Online Supplementary Document**).

We obtained publication counts from Web of Science and Scopus for selected diseases by searching for query terms with regular expressions in the title, abstract, or index. We conducted the search for a subset of the 67 infectious diseases since keywords in the database were not standardised, unlike MeSH terms. For example, diseases caused by the influenza virus and *Haemophilus influenzae* were difficult to discern in the search.

To count the publication numbers, publication types such as original research, reviews, and other formats were not distinguished, as all contribute to the visible research landscape. Publications addressing multiple diseases were counted once for each relevant disease, reflecting that such studies contribute to multiple research fields.

### Calculation of the BARI index

The BARI index was calculated as previously described [12]. Briefly, DALYs and publication counts were log-transformed, and a linear regression line was fitted to the log-transformed metrics after excluding data points more than two standard deviations from the mean in either DALYs or publication counts. Then, data points with residuals from the regression line exceeding two standard deviations were excluded as outliers, and another linear regression line was computed for the remaining data points. A BARI index is a residual from the second regression line, with standardisation to Z-scores with a mean of zero and a standard deviation of one, by dividing by the original standard deviation. We calculated the index for all aetiologies, including data points not used to compute the regression line. In essence, BARI captured whether a disease receives more or less research attention than would be expected given its burden. Positive values indicated relatively high research intensity, whereas negative values indicated relative neglect. As BARI indices were standardised, values >2 or<−2 were considered statistically significant.

The log transformation in the BARI computation described above was performed using base 10; however, changing the logarithm’s base does not affect the BARI index score. This is because logarithms with different bases differ only by a constant scaling factor, and therefore, using different bases does not affect the correlation or standardised residuals in the regression.

It should be noted that, because we used the regression as a descriptive reference rather than for causal inference or formal prediction, strict model assumptions were unnecessary. The purpose of the regression was not to demonstrate or test a strong linear relationship between DALYs and publication counts *per se*. Rather, we used the regression line derived from the main body of diseases, after excluding extreme values, as a reference to quantify how far each disease deviated from the general pattern. Without these exclusion steps, diseases that are themselves highly atypical could be treated as part of the ‘normal’ pattern, and because of their leverage, they could also pull the regression line toward themselves. This would reduce our ability to identify truly over- or under-studied diseases relative to the general trend. In other words, the BARI index should be interpreted as a measure relative to a regression line fitted to the non-outlying majority of diseases. This sensitivity is not a flaw incidental to the method but is closely related to the conceptual aim of BARI, namely, to detect diseases whose relative research intensity is markedly displaced from the common burden–publication structure.

We calculated the BARI index for the combined periods of 2016–2019 and 2020–2023, and for single-year window periods from 2016 to 2023. Regarding data on publication counts, the analyses were based on publication year rather than submission date because submission dates are not always reported and are therefore unsuitable for systematic analysis.

We also calculated the BARI index using mortality data or years of life lost (YLLs) instead of DALYs as a sensitivity analysis. In addition, we calculated the BARI index at the country level for 16 countries purposively selected to provide broad geographic coverage across Africa, the Middle East/North Africa, South Asia, East/Southeast Asia, Europe, Latin America, North America, and Oceania. In the country-level analyses, DALYs or publication counts are zero for some cases. Diseases with publications but zero DALYs were assigned the score of 4.9, while those with nonzero DALYs but no publications were assigned the score of −4.9.

### Statistical tests

We compared the BARI index scores in 2016–2019 and 2020–2023 between NTDs and non-NTDs. Also, single-year BARI indices were examined for individual diseases and compared between index scores from 2016 to 2019 (pre-COVID-19 pandemic) and those from 2020 to 2023 (post-pandemic). Because the statistical comparisons did not evaluate an absolute BARI index *per se* but tested differences between two time-period groups, the results can be statistically significant even if the BARI index at a single time point is not significantly high or low. We used the nonparametric Mann-Whitney U test to compare two independent groups, or the Wilcoxon signed-rank test for paired comparisons, because the Shapiro-Wilk test indicated departures from normality in some comparisons.

To evaluate whether the temporal change differed between NTDs and non-NTDs, we performed a repeated-measures analysis of variance and examined the group-by-time interaction. The difference in the proportion of diseases with significantly high or low BARI index and the difference in the proportion of diseases for which the BARI index decreased from 2016–2019 to 2020–2023 were tested between NTDs and non-NTDs using Fisher exact test. We performed statistical comparisons for 66 infectious diseases, excluding COVID-19. We considered *P*-values <0.05 statistically significant.

All data management and statistical analyses were conducted using Excel, version 2602 (Microsoft Corporation, Redmond, Washington, USA) and *R*, version 4.3.2 (R Core Team, Vienna, Austria). A tool to calculate the BARI index and code for statistical tests are available in a GitHub repository.

## RESULTS

**Figure 1:**
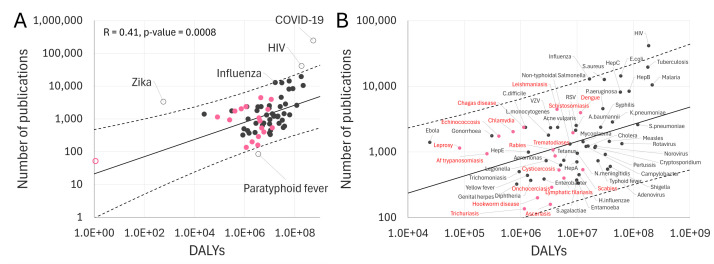
Correlation between disease burden and research output. **Panel A.** Sum of DALYs from 2020 to 2023 to represent disease burden and publication counts in the same period to represent research output for 67 infectious diseases were plotted, and a linear regression line and its 95% confidence intervals were drawn for the log-transformed values, excluding outliers (see the Methods for details. r = 0.41, *P* = 0.0008). Open circles indicate outliers that were not used for computing the regression analysis. Diseases outside the 95% confidence intervals were considered to have significantly high or low research intensity based on their disease burden, and their names were listed in the panel. **Panel B.** A magnified figure of panel A is shown. The names of all diseases are described, regardless of whether their BARI index scores are statistically significant. NTDs are coloured red. COVID-19, Zika, and guinea worm disease are located outside Panel B.

To examine research intensity during the COVID-19 pandemic across 67 infectious diseases, including 18 NTDs, we analysed DALYs and the number of publications in 2020–2023 and found a statistically significant correlation between the log-transformed values of them (Pearson r = 0.41, *P* = 0.0008 (**Figure 1**). For this, we obtained the bibliometric data from MEDLINE *via* PubMed; however, the database may not be comprehensive. Therefore, we also checked publication data in Web of Science and Scopus, both of which cover not only English-language academic journals but also conference proceedings and books in various languages. Publication counts from the Web of Science and Scopus were always higher than those from MEDLINE (Figure S1 in the **Online Supplementary Document**). That must be due not only to the latter two databases having larger data sets, but also to articles whose main topic is not a disease of interest but that contain query terms somewhere being hit by the keyword search. Still, publication counts from the two databases were highly correlated with the results obtained from MEDLINE (r = 0.85, *P* < 0.0001 for Web of Science and r = 0.83, *P* < 0.0001 for Scopus). Since the MEDLINE search using MeSH terms can specifically identify articles that focus on the disease of interest and minimise retrieval bias due to terminological variation, we used MEDLINE data for further analyses. From a statistical perspective, differences in absolute publication counts across different databases do not substantially affect our analysis, provided the values are nearly proportional.

**Figure 2:**
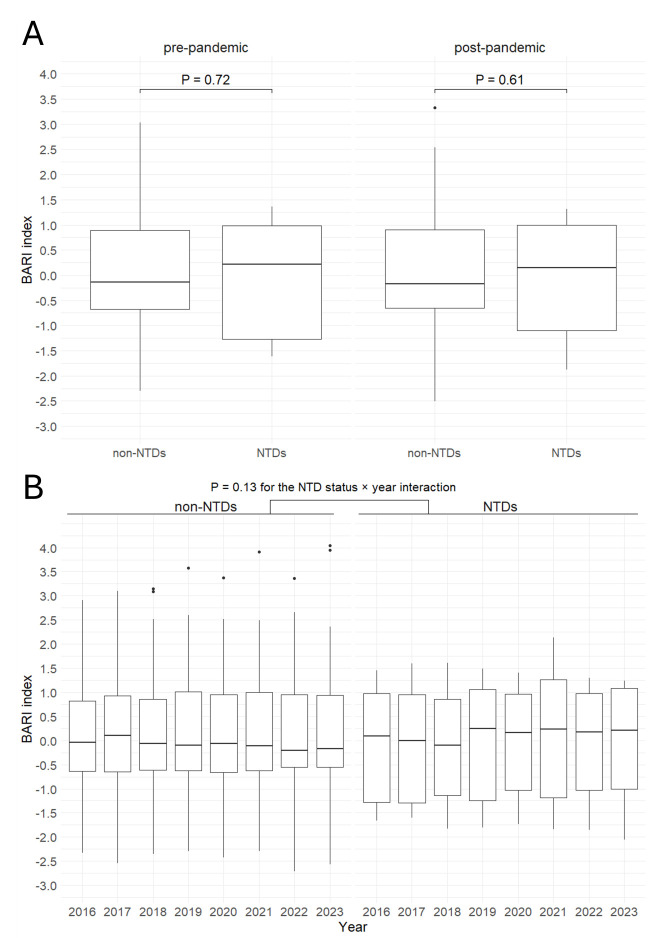
Change in relative research intensity from 2016 to 2023. **Panel A.** BARI index scores in pre-pandemic (2016–2019) and post-pandemic (2020–2023) periods are shown for non-NTDs and NTDs, separately. *P*-values were computed using the Mann-Whitney U test. **Panel B.** Single-year BARI indices were calculated and grouped by NTD status. The difference in longitudinal changes by year between non-NTDs and NTDs was tested by a repeated-measures ANOVA.

During 2020–2023, significantly high BARI was detected for COVID-19, HIV, influenza, and Zika. In contrast, paratyphoid fever, although not classified as an NTD, showed a significantly lower BARI. Importantly, there was no significant difference in the median BARI index scores between NTDs and non-NTDs (*P* = 0.61). This result indicates that classification as an NTD was not associated with lower relative research intensity during the pandemic period (**Figure 1**, **Figure 2**, Panel A).

**Figure 3:**
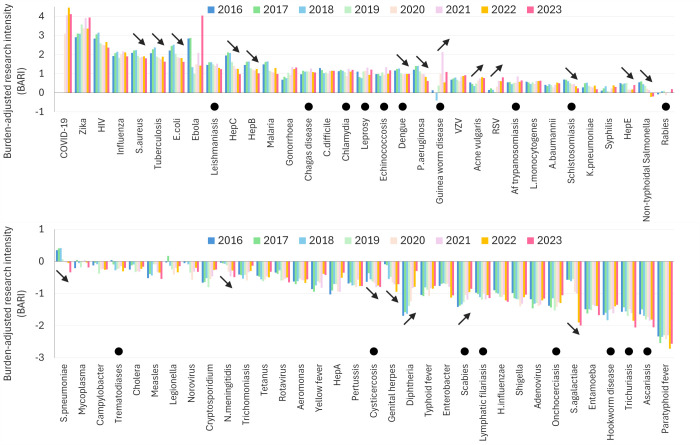
Relative research intensity of 67 infectious diseases from 2016 to 2023. Single-year BARI index scores for each of 67 infectious diseases are shown from 2016 to 2023. NTDs are indicated by black dots. Statistically significant differences between the periods before and after the COVID-19 pandemic (2016–2019 *vs.* 2020–2023), tested by the Mann-Whitney U test, are indicated by arrows in this figure. Diseases are sorted by BARI index score in 2020–2023. All data values and the results of statistical tests are available in Table S3 in the **Online Supplementary Document**.

We then tested changes in research intensity between the pre-COVID-19 pandemic (2016–2019) and post-pandemic (2020–2023) periods by calculating and comparing the BARI index scores for each disease each year. Among the 18 infectious NTDs analysed, three diseases, including cysticercosis, dengue, and schistosomiasis, showed a significant decrease in BARI during the post-pandemic period of 2020–2023. Among the 48 non-NTDs excluding COVID-19, 12 infectious diseases showed a significant decrease in the BARI index after the emergence of COVID-19. Thus, a decline in relative research intensity was observed for both NTDs and non-NTDs. Interestingly, the relative research intensity of all of the ‘big three’ infectious diseases, HIV, malaria, and tuberculosis, tended to decline, although not all changes were statistically significant (*P* = 0.057 for HIV, *P* = 0.057 for malaria, and *P* = 0.029 for tuberculosis) (**Figure 3**; Tables S3 and S4 in the **Online Supplementary Document**).

Statistical testing revealed no significant difference between NTDs and non-NTDs in the proportion of diseases experiencing a significant decrease in relative research intensity (n/N = 3/18, 16.7% NTDs *vs.* n/N = 12/48, 25.0% non-NTDs; *P* = 0.74; Fisher exact test), and longitudinal change between the two groups was not statistically different (*P* = 0.13) (**Figure 2**, Panel B). Those results suggest that NTDs, as a group, did not exhibit a disproportionate decline in relative research intensity during the COVID-19 pandemic.

To confirm the robustness of the findings, we also used mortality and YLLs as disease burden data instead of DALYs in calculating BARI. Still, neither the median BARI index scores nor the proportions of diseases that showed a significant decrease during the pandemic differed significantly between non-NTDs and NTDs (Table S5 in the **Online Supplementary Document**).

During the pandemic period of 2020–2023, seven diseases in the lowest quartile of relative research intensity were NTDs, whereas four NTDs were in the highest quartile. Although there was no statistically significant difference in the prevalence (n/N = 7/17, 41.2% in the lowest quartile *vs.* n/N = 4/17, 23.5% in the highest quartile; *P* = 0.46; Fisher exact test), several NTDs consistently occupied the lowest end of the BARI distribution throughout the study period, echoing patterns identified in earlier decades [12]. Thus, although the pandemic did not further worsen the relative position of NTDs as a category, low relative research intensity was observed for some NTDs and non-NTDs (**Figure 3**).

We then investigated the trend in the BARI index across 16 representative countries in different regions. The country-level analyses showed that BARI for NTDs was significantly higher than for non-NTDs in many countries, even during the 2020–2023 pandemic period. That is because many research-active countries have studied diseases that are not prevalent there [12]. The proportion of diseases with a significantly low BARI index or that experienced a significant decline in the index during the COVID-19 pandemic did not differ statistically between non-NTDs and NTDs in most countries. An exception was Kenya, where diseases with a significantly lower BARI index were more prevalent in non-NTDs than NTDs. A similar trend was observed for Nigeria and Indonesia, although not statistically significant (Table S5 in the **Online Supplementary Document**).

We also identified diseases whose research intensity increased during the COVID-19 pandemic. Among NTDs, the BARI index scores for guinea worm disease and scabies increased significantly globally. As for non-NTDs, acne vulgaris, diphtheria, and respiratory syncytial virus gained research attention in the period.

Lastly, we explored whether any factors were associated with changes in relative research intensity during the COVID-19 pandemic by grouping diseases based on their characteristics. Diseases of viral aetiology tend to receive higher research intensity, whether or not they are NTDs, in both the pre- and post-pandemic periods. However, even for viral diseases, the BARI did not increase significantly as a group during the COVID-19 pandemic. Research intensity for parasite-caused diseases was generally low in both the pre- and post-pandemic periods. When it comes to non-parasitic diseases, interestingly, research intensity was high for those designated as NTDs. Significant changes were not detected between the periods before and after the COVID-19 pandemic for either parasitic or non-parasitic diseases. There were no statistically significant differences in BARI between non-respiratory and respiratory diseases, in both the pre- and post-pandemic periods. The difference between the pre- and post-pandemic periods for respiratory diseases, as a group, was not statistically significant either (Figure S2 in the **Online Supplementary Document**).

## DISCUSSION

In this study, we investigated research intensity adjusted for disease burden across 67 infectious diseases before and after the COVID-19 pandemic. The absence of a marked decline in the BARI index for most NTDs indicates that research on these diseases was not disproportionately displaced by COVID-19. Our findings therefore do not support a broad conclusion that NTDs, as a category, received lower research intensity than other infectious diseases during the pandemic period. At the same time, the present study revealed heterogeneity across diseases. Not all NTDs received sufficient research attention during the COVID-19 pandemic. Cysticercosis, dengue, and schistosomiasis showed declining BARI indices, and ascariasis and trichuriasis remained at the lower end of the BARI distribution. A low level of research intensity for those diseases may simply reflect the persistence of entrenched structural constraints [10]. NTDs with low or declining BARI may attract limited research investment because they are associated with chronic health burdens in resource-limited settings and with limited commercial incentives for research and development [18–20].

In contrast, research on respiratory viral infections, such as influenza and respiratory syncytial virus, appears to have benefited indirectly from conceptual, technological, and infrastructural advances driven by COVID-19 research [21,22]. Shared methodologies, platforms for vaccine development, and heightened interest in the epidemiology of respiratory infectious diseases likely contributed to increased output in these areas (Figure S3 in the **Online Supplementary Document**). Yet, NTDs – many of which involve different transmission routes, pathogens, and intervention strategies – did not experience similar benefits. Even so, some technologies that emerged during the pandemic, including novel vaccine modalities such as mRNA-lipid nanoparticles and viral vectors, as well as next-generation sequencing for surveillance and epidemiological investigation, can also be applied to NTDs [23,24]. From this perspective, the pandemic can be seen as an opportunity to correct longstanding inequities in global health research.

Pandemic preparedness frameworks emphasise rapid detection, swift mobilisation, and surge capacity, all of which are essential for controlling novel threats [25]. Weak health systems, insufficient surveillance, and limited research capacity in regions affected by NTDs could undermine global preparedness for emerging pathogens [26]. Investment in NTD research should be viewed not as competing with pandemic preparedness, but as integral to it.

An important implication of our findings is that formal NTD designation alone may not be sufficient to capture diseases that receive limited research attention relative to their burden. Various heterogeneities exist among NTDs and may explain why some diseases showed a lower BARI index, while others did not. For example, dengue has substantial global visibility because of its vector-borne epidemic potential, expanding geographic range, and advances in immunological understanding, which have led to the development of promising vaccines [27]. In contrast, rabies has a distinct profile as a zoonotic disease with prevention tools available, but access to and implementation of these tools remain uneven. There are treatment options for many parasitic diseases, but environmental and logistical factors are still hurdles. These examples suggest that we should take the heterogeneity of NTDs into account to discuss the appropriate research efforts required. Analysis of NTDs as a single category may obscure important differences in geographic distribution, transmission route, outbreak potential, availability of interventions, and research ecosystems.

Furthermore, paratyphoid fever and amoebiasis (Entamoeba) were not classified as NTDs but showed markedly low relative research intensity. Likewise, some non-NTD diseases may share features commonly associated with neglected diseases, including concentration in resource-limited settings, limited commercial incentives for product development, and relatively low visibility in global research agendas [28]. In fact, we found that non-NTDs in certain countries receive less research attention than NTDs, and that non-parasitic non-NTDs attract less intensive research efforts than non-parasitic NTDs globally (Figure S2 and Table S5 in the **Online Supplementary Document**). Research prioritisation frameworks should consider not only formal designation of NTDs but also empirical indicators of research activity relative to their burden. Our approach using the BARI index can help identify such overlooked imbalances by providing an empirical measure of research activity relative to disease burden.

### Limitations

The use of the BARI index, based on scholarly publication counts and DALYs, provides a transparent way to assess relative research intensity [12]. However, publication counts may not capture the full picture of research activities, such as research quality and impact, funding intensity, capacity-building initiatives, patent applications, and progress in drug and vaccine development pipelines. Still, publications must most comprehensively reflect the entire research ecosystem in a measurable form across different diseases as output [29,30]. Our approach, which relies on a specific bibliometric database, may also have introduced regional and linguistic biases in publication coverage and overlooked non-indexed outputs. For example, grey literature was not included in our analysis. That is because we assumed that grey literature does not necessarily reflect research activities, though it can indicate stakeholder attention. While acknowledging its coverage limitations, we think our approach remains suitable for quantifying research intensity, as publications in internationally recognised academic journals capture research efforts across various sectors with visible, tangible social impact. Nevertheless, future work should aim to triangulate various bibliometric indicators with additional data sources [18,31,32]. Citation-based measures may also offer insight into the influence and uptake of research [10]. Integrating these diverse metrics would allow a more nuanced assessment of how research effort aligns with global health needs.

Another limitation is that, although DALYs from the GBD study represent the most authoritative global estimates, disease burden estimates for some NTDs remain uncertain due to limited surveillance data in certain regions. The selection of infectious diseases analysed in this study was limited by the availability of corresponding disease burden data (Tables S1 and S2 in the **Online Supplementary Document**). In addition, our analysis based on publication year did not account for delays between funding allocation, data collection and analysis, manuscript submission, and publication, which may be particularly relevant during the pandemic. The full impact of COVID-19 era research shifts may not become apparent for several years. As such, continued monitoring of research intensity beyond 2023 will be essential to determine whether the patterns observed here will persist or they just represent a transient equilibrium.

Finally, we concede that our approach has not been widely implemented or validated by other researchers. The BARI index was developed to compare how research output is distributed relative to disease burden across diseases and to identify outliers or temporal changes that warrant closer investigation [12]. For further discussion, we have developed an easy-to-use tool to calculate the BARI index using user-entered disease burden and research intensity data, which is available on GitHub. The tool is freely available, enabling other researchers to apply it to various situations.

## CONCLUSIONS

In conclusion, the COVID-19 pandemic did not substantially reduce the relative research intensity of NTDs as a category. Rather, our findings indicate that patterns of low or declining research intensity were heterogeneous, involving both NTDs and non-NTDs. Still, persistently low research intensity output for some NTDs during the COVID-19 pandemic reflects the persistence of entrenched research ecosystems rather than equitable or needs-driven prioritisation [10]. Transforming the structures that determine who leads research, which diseases are prioritised, and how resources are allocated across regions is required for addressing the issue [11].

Coincidentally, amendments to the International Health Regulations in 2025 emphasise the promotion of equity and solidarity [25,33]. This concept should be applied not only to public health but also to research activities. Global infectious disease research can be aligned with both urgent threats and enduring needs through responsible communication and deliberate policy action. As the importance of fair and globally inclusive infectious disease research continues to grow, ensuring equitable research efforts in truly neglected NTDs and other disproportionately neglected diseases will be essential for reducing global health disparities.

## Additional material


Online Supplementary Document

## Data Availability

**Data availability:** Data used in this study are available from publicly accessible platforms, including the GBD results tool and PubMed. The data sets used are also available upon reasonable request. A tool to calculate the BARI index and computer scripts for publication counts data retrieval and statistical tests are available at: https://github.com/yukifuruse1217/BARI_2026.
