# Supplementary material for: Research intensity for neglected tropical diseases and other infectious diseases before and after the COVID-19 pandemic
Source: J Glob Health. 2026 Aug 7;16:04255. doi: 10.7189/jogh.16.04255 (PMC13449551; doi:10.7189/jogh.16.04255)
Supplement: Supplementary file 1 [file jogh-16-04255-s001.pdf]

# Online Supplementary Document

## Table S1 List of analyzed diseases

| Disease/etiology | NTD | GBD classification (category name in the database) | Query for PubMed | Query for Web of Science and Scopus |
|---|---|---|---|---|
| A. baumannii | | Etiology (Acinetobacter baumannii) | "Acinetobacter baumannii"[MeSH Major Topic] OR "Acinetobacter Infections"[MeSH Major Topic] | acinetobacter* |
| Acne vulgaris | | Cause (Acne vulgaris) | "Acne Vulgaris"[MeSH Major Topic] OR "Propionibacterium acnes"[MeSH Major Topic] | acne* |
| Adenovirus | | Etiology (Adenovirus) | "Adenoviruses, Human"[MeSH Major Topic] OR "Adenovirus Infections, Human"[MeSH Major Topic] | adenovir* |
| Aeromonas | | Etiology (Aeromonas) | "Aeromonas"[MeSH Major Topic] | aeromonas* |
| Af trypanosomiasis | Yes | Cause (African trypanosomiasis) | "Trypanosomiasis, African"[MeSH Major Topic] OR "Trypanosoma brucei brucei"[MeSH Major Topic] OR "Trypanosoma brucei gambiense"[MeSH Major Topic] OR "Trypanosoma brucei rhodesiense"[MeSH Major Topic] | |
| Ascariasis | Yes | Cause (Ascariasis) | "Ascariasis"[MeSH Major Topic] OR "Ascaris lumbricoides"[MeSH Major Topic] | ascari* |
| C. difficile | | Etiology (Clostridium difficile) | "Clostridium difficile"[MeSH Major Topic] OR "Enterocolitis, Pseudomembranous"[MeSH Major Topic] | |
| Campylobacter | | Etiology (Campylobacter) | "Campylobacter Infections"[MeSH Major Topic] OR "Campylobacter"[MeSH Major Topic] | campylobacter* |
| Chagas disease | Yes | Cause (Chagas disease) | "Chagas Disease"[MeSH Major Topic] OR "Trypanosoma cruzi"[MeSH Major Topic] | chagas* |
| Chlamydia | Yes | Cause (Chlamydial infection) | "Chlamydia"[MeSH Major Topic] OR "Chlamydia Infections"[MeSH Major Topic] OR "Trachoma"[MeSH Major Topic] OR "Chlamydia trachomatis"[MeSH Major Topic] | chlamydia* |
| Cholera | | Etiology (Cholera) | "Cholera"[MeSH Major Topic] OR "Vibrio cholerae"[MeSH Major Topic] | cholera* |
| COVID-19 | | Cause (COVID-19) | "SARS-CoV-2"[MeSH Major Topic] OR "COVID-19"[MeSH Major Topic] | |
| Cryptosporidium | | Etiology (Cryptosporidium) | "Cryptosporidiosis"[MeSH Major Topic] OR "Cryptosporidium"[MeSH Major Topic] | cryptosporidi* |
| Cysticercosis | Yes | Cause (Cysticercosis) | "Cysticercosis"[MeSH Major Topic] OR "Taenia solium"[MeSH Major Topic] | cysticercosis* |
| Dengue | Yes | Cause (Dengue) | "Dengue"[MeSH Major Topic] OR "Dengue Virus"[MeSH Major Topic] | dengue* |
| Diphtheria | | Cause (Diphtheria) | "Diphtheria"[MeSH Major Topic] OR "Corynebacterium diphtheriae"[MeSH Major Topic] | diphtheria* |
| E. coli | | Etiology (Escherichia coli, Enterotoxigenic E coli, Enteropathogenic E coli) | "Escherichia coli"[MeSH Major Topic] OR "Escherichia coli Infections"[MeSH Major Topic] | "escherichia coli*" |

| | | | | |
|---|---|---|---|---|
| Ebola | | Cause (Ebola) | "Hemorrhagic Fever, Ebola"[MeSH Major Topic] OR "Ebolavirus"[MeSH Major Topic] | ebola* |
| Echinococcosis | Yes | Cause (Cystic echinococcosis) | "Echinococcosis"[MeSH Major Topic] OR "Echinococcus"[MeSH Major Topic] | echinococc* |
| Entamoeba | | Etiology (Entamoeba) | "Dysentery, Amebic"[MeSH Major Topic] OR "Entamoeba histolytica"[MeSH Major Topic] | |
| Enterobacter | | Etiology (Enterobacter spp) | "Enterobacter"[MeSH Major Topic] | enterobacter* |
| Genital herpes | | Cause (Genital herpes) | "Herpes Genitalis"[MeSH Major Topic] OR "Herpesvirus 2, Human"[MeSH Major Topic] | |
| Gonorrhea | | Cause (Gonococcal infection) | "Gonorrhea"[MeSH Major Topic] OR "Neisseria gonorrhoeae"[MeSH Major Topic] | gonorrh* |
| Guinea worm disease | Yes | Cause (Guinea worm disease) | "Dracunculiasis"[MeSH Major Topic] OR "Dracunculus Nematode"[MeSH Major Topic] | dracuncul* |
| H. influenzae | | Etiology (Haemophilus influenzae) | "Haemophilus influenzae type b"[MeSH Major Topic] OR "Haemophilus Infections"[MeSH Major Topic] | |
| Hep A | | Cause (Acute hepatitis A) | "Hepatitis A"[MeSH Major Topic] OR "Hepatitis A virus"[MeSH Major Topic] | "hepatitis A*" |
| Hep B | | Cause (Total burden related to hepatitis B) | "Hepatitis B"[MeSH Major Topic] OR "Hepatitis B virus"[MeSH Major Topic] | "hepatitis B*" |
| Hep C | | Cause (Total burden related to hepatitis C) | "Hepatitis C"[MeSH Major Topic] OR "Hepacivirus"[MeSH Major Topic] | "hepatitis C*" |
| Hep E | | Cause (Acute hepatitis E) | "Hepatitis E"[MeSH Major Topic] OR "Hepatitis E virus"[MeSH Major Topic] | "hepatitis E*" |
| HIV | | Cause (HIV/AIDS resulting in other diseases, HIV/AIDS - Drug-susceptible Tuberculosis, HIV/AIDS - Multidrug-resistant Tuberculosis without extensive drug resistance, HIV/AIDS - Extensively drug-resistant Tuberculosis) | "HIV"[MeSH Major Topic] OR "HIV Infections"[MeSH Major Topic] | |
| Hookworm disease | Yes | Cause (Hookworm disease) | "Hookworm Infections"[MeSH Major Topic] OR "Ancylostoma"[MeSH Major Topic] | |
| Influenza | | Etiology (Influenza) | "Influenza, Human"[MeSH Major Topic] OR "Influenzavirus A"[MeSH Major Topic] OR "Influenzavirus B"[MeSH Major Topic] OR "Influenzavirus C"[MeSH Major Topic] | |

| | | | | |
|---|---|---|---|---|
| K. pneumoniae | | Etiology (Klebsiella pneumoniae) | "Klebsiella pneumoniae"[MeSH Major Topic] OR "Klebsiella Infections"[MeSH Major Topic] | klebsiella* |
| L. monocytogenes | | Etiology (Listeria monocytogenes) | "Listeria monocytogenes"[MeSH Major Topic] OR "Meningitis, Listeria"[MeSH Major Topic] OR "Listeriosis"[MeSH Major Topic] | listeri* |
| Legionella | | Etiology (Legionella spp) | "Legionella"[MeSH Major Topic] OR "Legionellosis"[MeSH Major Topic] | legionell* |
| Leishmaniasis | Yes | Cause (Visceral leishmaniasis) | "Leishmaniasis"[MeSH Major Topic] OR "Leishmania"[MeSH Major Topic] | leishmania* |
| Leprosy | Yes | Cause (Leprosy) | "Leprosy"[MeSH Major Topic] OR "Mycobacterium leprae"[MeSH Major Topic] | leprosy* |
| Lymphatic filariasis | Yes | Cause (Lymphatic filariasis) | "Elephantiasis, Filarial"[MeSH Major Topic] OR "Brugia"[MeSH Major Topic] OR "Wuchereria bancrofti"[MeSH Major Topic] | |
| Malaria | | Cause (Malaria) | "Malaria"[MeSH Major Topic] OR "Plasmodium"[MeSH Major Topic] | malaria* |
| Measles | | Cause (Measles) | "Measles"[MeSH Major Topic] OR "Measles virus"[MeSH Major Topic] | measles* |
| Mycoplasma | | Etiology (Mycoplasma) | "Mycoplasma pneumoniae"[MeSH Major Topic] OR "Mycoplasma Infections"[MeSH Major Topic] | mycoplasma* |
| N. meningitidis | | Etiology (Neisseria meningitidis) | "Meningococcal Infections"[MeSH Major Topic] OR "Neisseria meningitidis"[MeSH Major Topic] | |
| Non-typhoidal Salmonella | | Etiology (Non-typhoidal Salmonella) | "Salmonella Food Poisoning"[MeSH Major Topic] OR "Salmonella enteritidis"[MeSH Major Topic] OR "Salmonella typhimurium"[MeSH Major Topic] OR "Salmonella arizonae"[MeSH Major Topic] | |
| Norovirus | | Etiology (Norovirus) | "Norovirus"[MeSH Major Topic] OR "Caliciviridae Infections"[MeSH Major Topic] | norovir* |
| Onchocerciasis | Yes | Cause (Onchocerciasis) | "Onchocerciasis"[MeSH Major Topic] OR "Onchocerca"[MeSH Major Topic] | onchocerc* |
| P. aeruginosa | | Etiology (Pseudomonas aeruginosa) | "Pseudomonas aeruginosa"[MeSH Major Topic] OR "Pseudomonas Infections"[MeSH Major Topic] | pseudomonas* |
| Paratyphoid fever | | Cause (Paratyphoid fever) | "Paratyphoid Fever"[MeSH Major Topic] OR "Salmonella paratyphi A"[MeSH Major Topic] OR "Salmonella paratyphi B"[MeSH Major Topic] OR "Salmonella paratyphi C"[MeSH Major Topic] | paratyph* |
| Pertussis | | Cause (Pertussis) | "Whooping Cough"[MeSH Major Topic] OR "Bordetella pertussis"[MeSH Major Topic] | pertussis* |
| Rabies | Yes | Cause (Rabies) | "Rabies"[MeSH Major Topic] OR "Rabies virus"[MeSH Major Topic] | rabies* |
| Rotavirus | | Etiology (Rotavirus) | "Rotavirus"[MeSH Major Topic] OR "Rotavirus Infections"[MeSH Major Topic] | rotavir* |
| RSV | | Etiology (Respiratory syncytial virus) | "Respiratory Syncytial Viruses"[MeSH Major Topic] OR "Respiratory Syncytial Virus, Human"[MeSH Major Topic] OR "Respiratory Syncytial Virus Infections"[MeSH Major Topic] | |
| S. agalactiae | | Etiology (Group B streptococcus) | "Streptococcus agalactiae"[MeSH Major Topic] | |
| S. aureus | | Etiology (Staphylococcus aureus) | "Staphylococcus aureus"[MeSH Major Topic] OR "Staphylococcal Infections"[MeSH Major Topic] | staphylococc* |

| | | | | |
|---|---|---|---|---|
| S. pneumoniae | | Etiology (Streptococcus pneumoniae) | "Pneumococcal Infections"[MeSH Major Topic] OR "Streptococcus pneumoniae"[MeSH Major Topic] | |
| Scabies | Yes | Cause (Scabies) | "Scabies"[MeSH Major Topic] OR "Sarcoptes scabiei"[MeSH Major Topic] | scabi* |
| Schistosomiasis | Yes | Schistosomiasis | "Schistosomiasis"[MeSH Major Topic] OR "Schistosoma"[MeSH Major Topic] | schistosom* |
| Shigella | | Etiology (Shigella) | "Dysentery, Bacillary"[MeSH Major Topic] OR "Shigella"[MeSH Major Topic] | shigella* |
| Syphilis | | Cause (Syphilis) | "Syphilis"[MeSH Major Topic] OR "Treponema pallidum"[MeSH Major Topic] | syphilis* |
| Tetanus | | Cause (Tetanus) | "Tetanus"[MeSH Major Topic] OR "Clostridium tetani"[MeSH Major Topic] | tetan* |
| Trematodiases | Yes | Cause (Food-borne trematodiases) | "Clonorchiasis"[MeSH Major Topic] OR "Opisthorchiasis"[MeSH Major Topic] OR "Fascioliasis"[MeSH Major Topic] OR "Paragonimiasis"[MeSH Major Topic] OR "Clonorchis sinensis"[MeSH Major Topic] OR "Opisthorchis"[MeSH Major Topic] OR "Fasciola"[MeSH Major Topic] OR "Paragonimus"[MeSH Major Topic] | |
| Trichomoniasis | | Cause (Trichomoniasis) | "Trichomonas Infections"[MeSH Major Topic] OR "Trichomonas"[MeSH Major Topic] | trichomonas* |
| Trichuriasis | Yes | Cause (Trichuriasis) | "Trichuriasis"[MeSH Major Topic] OR "Trichuris"[MeSH Major Topic] | trichuri* |
| Tuberculosis | | Cause (Drug-susceptible tuberculosis, Multidrug-resistant tuberculosis without extensive drug resistance, Extensively drug-resistant tuberculosis, Latent tuberculosis infection) | "Tuberculosis"[MeSH Major Topic] OR "Mycobacterium tuberculosis"[MeSH Major Topic] | tuberculosis* |
| Typhoid fever | | Cause (Typhoid fever) | "Typhoid Fever"[MeSH Major Topic] OR "Salmonella typhi"[MeSH Major Topic] | |
| VZV | | Cause (Varicella and herpes zoster) | "Herpesvirus 3, Human"[MeSH Major Topic] OR "Varicella Zoster Virus Infection"[MeSH Major Topic] | |
| Yellow fever | | Cause (Yellow fever) | "Yellow Fever"[MeSH Major Topic] OR "Yellow Fever Virus"[MeSH Major Topic] | "yellow fever" |
| Zika | | Cause (Zika virus) | "Zika Virus"[MeSH Major Topic] OR "Zika Virus Infection"[MeSH Major Topic] | zika* |

Cause: cause of death or injury

All query combinations and R code for query search are available at: https://github.com/yukifuruse1217/BARI_2026

## Table S2 List of neglected tropical diseases

| Disease | WHO | CDC | Inclusion in this study (name) | Comment (reason for exclusion, etc.) |
|---|---|---|---|---|
| Buruli ulcer | Yes | Yes | No | No GBD data |
| Chagas disease | Yes | Yes | Yes (Chagas disease) | |
| chikungunya | Yes | Yes | No | No GBD data |
| dengue | Yes | Yes | Yes (Dengue) | |
| dracunculiasis (guinea worm disease) | Yes | Yes | Yes (Guinea worm disease) | |
| echinococcosis | Yes | Yes | Yes (Echinococcosis | |
| Fascioliasis | Yes? | Yes | Yes (Trematodiases) | Fascioliasis and foodborne trematodiases are not separated |
| foodborne trematodiases | Yes | Yes | Yes (Trematodiases) | Fascioliasis and foodborne trematodiases are not separated |
| human African trypanosomiasis | Yes | Yes | Yes (Af trypanosomiasis) | |
| leishmaniasis | Yes | Yes | Yes (Leishmaniasis) | |
| leprosy | Yes | Yes | Yes (Leprosy) | |
| lymphatic filariasis | Yes | Yes | Yes (Lymphatic filariasis) | |
| mycetoma, chromoblastomycosis and other deep mycoses | Yes | Yes | No | No GBD data |
| noma | Yes | No | No | No GBD data, absence of specific etiology |
| onchocerciasis | Yes | Yes | Yes (Onchocerciasis) | |
| rabies | Yes | Yes | Yes (Rabies) | |
| scabies and other ectoparasitoses | Yes | No | Yes (Scabies) | |
| schistosomiasis | Yes | Yes | Yes (Schistosomiasis) | |
| soil-transmitted helminthiases | Yes | Yes | Yes (Trichuriasis, Ascariasis, Hookworm disease) | |
| snakebite envenoming | Yes | No | No | No GBD data, not infectious disease |
| taeniasis/cysticercosis | Yes | No | Yes (Cysticercosis) | |
| trachoma | Yes | Yes | Yes (Chlamydia) | |
| yaws | Yes | Yes | No | No GBD data |

## Table S3 Single-year BARI index and comparison between pre- and post-COVID-19 pandemic periods

| Disease | BARI | | | | | | | | Median before pandemic | Median after pandemic | P value by U test |
|---|---|---|---|---|---|---|---|---|---|---|---|
| | 2016 | 2017 | 2018 | 2019 | 2020 | 2021 | 2022 | 2023 | | | |
| A.baumannii | 0.42 | 0.36 | 0.45 | 0.40 | 0.29 | 0.42 | 0.53 | 0.50 | 0.41 | 0.46 | 0.49 |
| Acne vulgaris | 0.54 | 0.47 | 0.36 | 0.49 | 0.64 | 0.75 | 0.82 | 0.77 | 0.48 | 0.76 | **0.029** |
| Adenovirus | -1.18 | -1.46 | -1.32 | -1.29 | -1.39 | -1.35 | -1.21 | -1.16 | -1.31 | -1.28 | 0.89 |
| Aeromonas | -0.63 | -0.71 | -0.61 | -0.53 | -0.58 | -0.59 | -0.67 | -0.56 | -0.62 | -0.59 | 0.69 |
| Af trypanosomiasis | 0.55 | 0.56 | 0.45 | 0.52 | 0.54 | 0.86 | 0.57 | 0.65 | 0.53 | 0.61 | 0.11 |
| Ascariasis | -1.65 | -1.49 | -1.69 | -1.81 | -1.74 | -1.85 | -1.81 | -2.05 | -1.67 | -1.83 | 0.057 |
| C.difficile | 1.29 | 1.19 | 1.03 | 1.06 | 1.01 | 1.15 | 1.15 | 1.15 | 1.13 | 1.15 | 0.69 |
| COVID-19 | – | – | – | – | 3.09 | 4.05 | 4.46 | 4.12 | – | 4.09 | – |
| Campylobacter | -0.12 | -0.04 | -0.09 | -0.38 | -0.25 | -0.23 | -0.26 | -0.25 | -0.11 | -0.25 | 0.34 |
| Chagas disease | 0.97 | 1.15 | 1.11 | 1.12 | 1.11 | 1.27 | 1.08 | 1.06 | 1.11 | 1.10 | 1.00 |
| Chlamydia | 1.13 | 1.20 | 1.16 | 1.08 | 0.88 | 1.25 | 1.11 | 1.20 | 1.14 | 1.15 | 1.00 |
| Cholera | -0.25 | -0.12 | -0.09 | -0.33 | -0.31 | -0.32 | -0.23 | -0.16 | -0.19 | -0.27 | 0.69 |
| Cryptosporidium | -0.66 | -0.64 | -0.52 | -0.80 | -0.55 | -0.46 | -0.27 | -0.25 | -0.65 | -0.36 | 0.057 |
| Cysticercosis | -0.63 | -0.37 | -0.56 | -0.62 | -0.67 | -0.80 | -0.75 | -0.80 | -0.59 | -0.77 | **0.029** |
| Dengue | 1.17 | 1.25 | 1.27 | 1.03 | 0.96 | 1.00 | 0.99 | 0.99 | 1.21 | 0.99 | **0.029** |
| Diphtheria | -1.69 | -1.58 | -1.63 | -1.39 | -1.24 | -0.83 | -0.79 | -0.30 | -1.61 | -0.81 | **0.029** |
| E.coli | 2.21 | 2.45 | 2.52 | 2.05 | 1.87 | 1.83 | 1.82 | 1.62 | 2.33 | 1.82 | **0.029** |
| Ebola | 2.84 | 2.87 | 1.34 | 1.01 | 1.49 | 2.09 | 1.42 | 4.04 | 2.09 | 1.79 | 0.69 |
| Echinococcosis | 0.98 | 0.99 | 0.89 | 1.07 | 0.95 | 1.34 | 1.00 | 1.18 | 0.98 | 1.09 | 0.34 |
| Entamoeba | -1.49 | -1.62 | -1.49 | -1.52 | -1.35 | -1.39 | -1.39 | -1.67 | -1.50 | -1.39 | 0.34 |
| Enterobacter | -0.76 | -0.70 | -0.68 | -0.70 | -0.69 | -0.79 | -1.13 | -1.06 | -0.70 | -0.93 | 0.20 |
| Genital herpes | -0.09 | -0.12 | -0.55 | -0.48 | -0.66 | -0.71 | -0.95 | -0.71 | -0.30 | -0.71 | **0.029** |
| Gonorrhoea | 0.68 | 0.84 | 0.77 | 1.05 | 0.93 | 1.35 | 1.24 | 1.33 | 0.80 | 1.28 | 0.057 |
| Guinea worm disease | 0.13 | 0.01 | -0.41 | 0.38 | 1.01 | 2.13 | 0.54 | 1.09 | 0.07 | 1.05 | **0.029** |
| H.influenzae | -0.90 | -0.98 | -1.00 | -1.10 | -1.11 | -1.04 | -1.21 | -1.26 | -0.99 | -1.16 | 0.057 |
| HIV | 2.84 | 3.06 | 3.15 | 2.60 | 2.52 | 2.50 | 2.66 | 2.36 | 2.95 | 2.51 | 0.057 |
| HepA | -1.03 | -0.90 | -0.70 | -0.71 | -0.93 | -0.95 | -0.51 | -0.35 | -0.80 | -0.72 | 0.69 |

| | | | | | | | | | | | |
|---|---|---|---|---|---|---|---|---|---|---|---|
| HepB | 1.46 | 1.63 | 1.64 | 1.28 | 1.22 | 1.17 | 1.25 | 1.02 | 1.54 | 1.19 | **0.029** |
| HepC | 1.95 | 2.11 | 2.08 | 1.61 | 1.41 | 1.26 | 1.25 | 0.98 | 2.02 | 1.26 | **0.029** |
| HepE | 0.51 | 0.44 | 0.49 | 0.52 | 0.18 | 0.15 | 0.17 | 0.41 | 0.50 | 0.18 | **0.029** |
| Hookworm disease | -1.67 | -1.60 | -1.83 | -1.51 | -1.48 | -1.61 | -1.40 | -1.35 | -1.63 | -1.44 | 0.11 |
| Influenza | 1.93 | 2.08 | 2.15 | 1.84 | 2.03 | 2.17 | 2.11 | 1.91 | 2.00 | 2.07 | 0.69 |
| K.pneumoniae | 0.28 | 0.50 | 0.53 | 0.36 | 0.32 | 0.28 | 0.37 | 0.19 | 0.43 | 0.30 | 0.20 |
| L.monocytogenes | 0.58 | 0.54 | 0.46 | 0.55 | 0.46 | 0.62 | 0.61 | 0.63 | 0.54 | 0.62 | 0.20 |
| Legionella | -0.03 | 0.17 | -0.14 | -0.25 | -0.41 | -0.20 | -0.34 | -0.15 | -0.08 | -0.27 | 0.11 |
| Leishmaniasis | 1.46 | 1.59 | 1.60 | 1.49 | 1.41 | 1.52 | 1.30 | 1.24 | 1.54 | 1.35 | 0.11 |
| Leprosy | 1.10 | 0.82 | 0.77 | 1.12 | 1.15 | 1.32 | 0.94 | 1.21 | 0.96 | 1.18 | 0.11 |
| Lymphatic filariasis | -0.97 | -1.02 | -1.11 | -1.19 | -0.99 | -1.18 | -1.05 | -1.14 | -1.06 | -1.10 | 0.89 |
| Malaria | 1.49 | 1.62 | 1.64 | 1.15 | 1.10 | 1.07 | 1.30 | 0.99 | 1.55 | 1.08 | 0.057 |
| Measles | -0.52 | -0.39 | -0.44 | -0.09 | -0.10 | -0.34 | -0.35 | -0.54 | -0.42 | -0.34 | 0.89 |
| Mycoplasma | -0.21 | 0.05 | -0.05 | -0.18 | -0.03 | 0.04 | -0.03 | -0.18 | -0.12 | -0.03 | 0.69 |
| N.meningitidis | -0.04 | -0.06 | -0.07 | -0.12 | -0.32 | -0.47 | -0.28 | -0.49 | -0.07 | -0.39 | **0.029** |
| Non-typhoidal Salmonella | 0.55 | 0.60 | 0.45 | 0.36 | 0.21 | 0.15 | -0.22 | -0.19 | 0.50 | -0.02 | **0.029** |
| Norovirus | -0.05 | -0.01 | -0.09 | -0.35 | -0.57 | -0.32 | -0.20 | -0.32 | -0.07 | -0.32 | 0.20 |
| Onchocerciasis | -1.38 | -1.43 | -1.15 | -1.53 | -1.41 | -1.27 | -1.30 | -1.05 | -1.41 | -1.28 | 0.34 |
| P.aeruginosa | 1.21 | 1.40 | 1.40 | 1.12 | 0.99 | 0.98 | 0.83 | 0.62 | 1.30 | 0.90 | **0.029** |
| Paratyphoid fever | -2.34 | -2.54 | -2.35 | -2.30 | -2.42 | -2.30 | -2.72 | -2.56 | -2.34 | -2.49 | 0.34 |
| Pertussis | -0.69 | -0.66 | -0.75 | -0.74 | -0.80 | -0.57 | -0.77 | -0.76 | -0.71 | -0.77 | 0.34 |
| RSV | 0.15 | 0.23 | 0.16 | 0.04 | 0.32 | 0.63 | 0.61 | 0.81 | 0.15 | 0.62 | **0.029** |
| Rabies | -0.08 | -0.03 | 0.09 | 0.11 | -0.13 | 0.02 | 0.00 | 0.19 | 0.03 | 0.01 | 1.00 |
| Rotavirus | -0.35 | -0.38 | -0.28 | -0.59 | -0.59 | -0.54 | -0.50 | -0.65 | -0.37 | -0.56 | 0.20 |
| S.agalactiae | -0.57 | -0.57 | -0.62 | -0.55 | -0.95 | -1.00 | -1.90 | -2.00 | -0.57 | -1.45 | **0.029** |
| S.aureus | 2.09 | 2.21 | 2.23 | 1.93 | 1.84 | 1.88 | 1.91 | 1.80 | 2.15 | 1.86 | **0.029** |
| S.pneumoniae | 0.35 | 0.41 | 0.42 | 0.07 | 0.01 | -0.03 | -0.05 | -0.34 | 0.38 | -0.04 | **0.029** |
| Scabies | -1.42 | -1.38 | -1.34 | -1.27 | -1.05 | -1.19 | -0.96 | -0.85 | -1.36 | -1.00 | **0.029** |
| Schistosomiasis | 0.69 | 0.69 | 0.61 | 0.50 | 0.45 | 0.45 | 0.35 | 0.23 | 0.65 | 0.40 | **0.029** |
| Shigella | -0.98 | -1.15 | -1.17 | -1.19 | -1.40 | -1.32 | -1.11 | -1.03 | -1.16 | -1.22 | 0.69 |
| Syphilis | 0.11 | 0.23 | 0.34 | 0.09 | 0.07 | 0.23 | 0.39 | 0.31 | 0.17 | 0.27 | 0.89 |

| | | | | | | | | | | | |
|---|---|---|---|---|---|---|---|---|---|---|---|
| Tetanus | -0.45 | -0.47 | -0.56 | -0.62 | -0.57 | -0.47 | -0.49 | -0.32 | -0.52 | -0.48 | 0.69 |
| Trematodiases | 0.04 | -0.09 | -0.28 | -0.24 | -0.22 | -0.15 | -0.31 | -0.20 | -0.17 | -0.21 | 0.69 |
| Trichomoniasis | -0.41 | -0.43 | -0.54 | -0.42 | -0.59 | -0.34 | -0.30 | -0.14 | -0.42 | -0.32 | 0.34 |
| Trichuriasis | -1.57 | -1.43 | -1.56 | -1.70 | -1.49 | -1.61 | -1.85 | -2.06 | -1.56 | -1.73 | 0.34 |
| Tuberculosis | 2.07 | 2.28 | 2.37 | 1.90 | 1.84 | 1.75 | 1.89 | 1.63 | 2.18 | 1.80 | **0.029** |
| Typhoid fever | -1.04 | -1.08 | -0.81 | -0.88 | -1.08 | -0.84 | -0.85 | -0.76 | -0.96 | -0.84 | 0.49 |
| VZV | 0.69 | 0.75 | 0.80 | 0.68 | 0.61 | 0.85 | 0.88 | 0.92 | 0.72 | 0.87 | 0.34 |
| Yellow fever | -0.86 | -0.94 | -0.75 | -0.62 | -0.67 | -0.82 | -0.38 | -0.42 | -0.80 | -0.55 | 0.20 |
| Zika | 2.91 | 3.10 | 3.09 | 3.58 | 3.38 | 3.91 | 3.36 | 3.94 | 3.10 | 3.64 | 0.11 |

P values less than 0.05 are bold.

**Table S4 List of diseases with significant change in BARI between pre- and post-COVID-19 pandemic periods**

| | non-NTDs | NTDs |
|---|---|---|
| Increase | Acne vulgaris, Diphtheria, RSV | Guinea worm disease, Scabies |
| Decrease | *E.coli*, Genital herpes, HepB, HepC, HepE, *N.meningitidis*, Non-typhoidal Salmonella, *P.aeruginosa*, *S.agalactiae*, *S.aureus*, *S.pneumoniae*, Tuberculosis | Cysticercosis, Dengue, Schistosomiasis |

## Table S5 Results of BARI calculation using different metrics or at country level

| Region/country | Disease burden metrics | Median BARI for non-NTDs in 2020-2023 (Q1, Q3) | Median BARI for NTDs in 2020-2023 (Q1, Q3) | P value by U test | BARI < -2 among non-NTDs in 2020–2023 (%) | BARI < -2 among NTDs in 2020–2023 (%) | NTD with BARI < -2 | P value by Fisher's exact test | Number of non-NTDs with significant decrease in BAR during the pandemic (%) | Number of NTDs with significant decrease in BAR during the pandemic (%) | NTD with significant BARI decrease | P value by Fisher's exact test |
|---|---|---|---|---|---|---|---|---|---|---|---|---|
| Global | DALYs | -0.17 (-0.66, 0.90) | 0.15 (-1.10, 0.99) | 0.61 | 1/48 (2.1%) | 0/18 (0%) | | 1.00 | 12/48 (25.0%) | 3/18 (16.7%) | Cysticercosis, Dengue, Schistosomiasis | 0.74 |
| Global | Mortality* | -0.36 (-0.85, 0.72) | 0.72 (0.01, 1.05) | 0.13 | 1/45 (2.2%) | 0/10 (0.0%) | | 1.00 | 16/45 (35.6%) | 2/10 (20%) | Dengue, Schistosomiasis | 0.47 |
| Global | YLLs* | -0.21 (-0.85, 0.86) | 0.67 (-0.08, 0.96) | 0.24 | 1/45 (2.2%) | 0/10 (0.0%) | | 1.00 | 16/45 (35.6%) | 2/10 (20%) | Dengue, Schistosomiasis | 0.47 |
| South Africa | DALYs | 0.13 (-0.49, 0.91) | 1.56 (0.17, 4.90) | **0.018** | 2/48 (4.2%) | 0/18 (0.0%) | | 1.00 | 0/48 (0%) | 0/18 (0%) | | 1.00 |
| Nigeria | DALYs | -0.10 (-1.04, 0.88) | 1.00 (0.01, 1.43) | **0.016** | 5/48 (10.4%) | 0/18 (0%) | | 0.31 | 2/48 (4.2%) | 0/18 (0%) | | 1.00 |
| Kenya | DALYs | -0.19 (-1.53, 0.74) | 0.57 (-0.16, 1.64) | **0.025** | 8/48 (16.7%) | 0/18 (0%) | | **0.048** | 0/48 (0%) | 1/18 (5.6%) | Cysticercosis | 0.27 |
| Egypt | DALYs | -0.02 (-0.92, 0.73) | 0.97 (0.16, 2.64) | **0.0071** | 3/48 (6.2%) | 0/18 (0%) | | 0.55 | 2/48 (4.2%) | 1/18 (5.6%) | Schistosomiasis | 1.00 |
| Saudi Arabia | DALYs | 0.35 (-0.83, 0.86) | 3.31 (0.18, 4.90) | **0.0036** | 1/18 (5.6%) | 0/18 (0%) | Hookworm disease | 1.00 | 2/48 (4.2%) | 0/18 (0%) | | 1.00 |
| India | DALYs | 0.15 (-0.59, 0.85) | 1.50 (0.06, 4.90) | **0.021** | 1/48 (2.1%) | 1/18 (5.6%) | Trichuriasis | 0.46 | 3/48 (6.2%) | 0/18 (0%) | | 0.56 |
| Bangladesh | DALYs | 0.03 (-0.74, 1.03) | 0.35 (-0.22, 2.65) | 0.26 | 3/48 (6.2%) | 2/18 (11.1%) | | 0.60 | 1/48 (2.1%) | 0/18 (0%) | | 1.00 |
| Indonesia | DALYs | 0.12 (-0.85, 0.78) | 0.93 (-0.10, 2.82) | **0.036** | 4/48 (8.3%) | 0/18 (0%) | | 0.31 | 0/48 (0%) | 0/18 (0%) | | 1.00 |
| Japan | DALYs | 0.04 (-0.58, 0.98) | 2.21 (-0.12, 4.90) | **0.016** | 2/48 (4.2%) | 1/18 (5.6%) | Scabies | 1.00 | 3/48 (6.2%) | 0/18 (0%) | | 0.56 |
| China | DALYs | 0.35 (-0.38, 1.07) | 0.42 (-1.46, 4.24) | 0.89 | 0/48 (0%) | 1/18 (5.6%) | Scabies | 0.26 | 5/48 (10.4%) | 1/18 (5.6%) | Schistosomiasis | 1.00 |
| Germany | DALYs | 0.15 (-0.72, 1.00) | 4.90 (1.17, 4.90) | **<0.0001** | 1/48 (2.1%) | 0/18 (0%) | | 1.00 | 3/48 (6.2%) | 1/18 (5.6%) | Chlamydia | 1.00 |
| United Kingdom | DALYs | 0.13 (-0.68, 1.20) | 4.90 (0.94, 4.90) | **0.00012** | 2/48 (4.2%) | 0/18 (0%) | | 1.00 | 8/48 (16.7%) | 0/18 (0%) | | 0.095 |
| Brazil | DALYs | -0.04 (-0.33, 0.54) | 0.55 (-0.69, 1.87) | 0.19 | 3/48 (6.2%) | 1/18 (5.6%) | Scabies | 1.00 | 5/48 (10.4%) | 0/18 (0%) | | 0.31 |
| Mexico | DALYs | -0.25 (-0.74, 0.68) | 0.34 (0.00, 1.83) | 0.11 | 2/48 (4.2%) | 1/18 (5.6%) | Hookworm disease | 1.00 | 2/48 (4.2%) | 1/18 (5.6%) | Cysticercosis | 1.00 |
| United States | DALYs | 0.34 (-0.5, 1.02) | 4.23 (0.78, 4.90) | **0.0087** | 0/48 (0%) | 1/18 (5.6%) | Scabies | 0.26 | 4/48 (8.3%) | 0/18 (0%) | | 0.57 |
| Australia | DALYs | 0.30 (-0.56, 1.06) | 4.90 (0.15, 4.90) | **0.004** | 0/48 (0%) | 0/18 (0%) | | 1.00 | 1/48 (2.1%) | 2/18 (11.1%) | Chlamydia, Echinococcosis | 0.18 |

* Non-fatal diseases were excluded from analyses using mortality or YLLs.

P values less than 0.05 are bold.

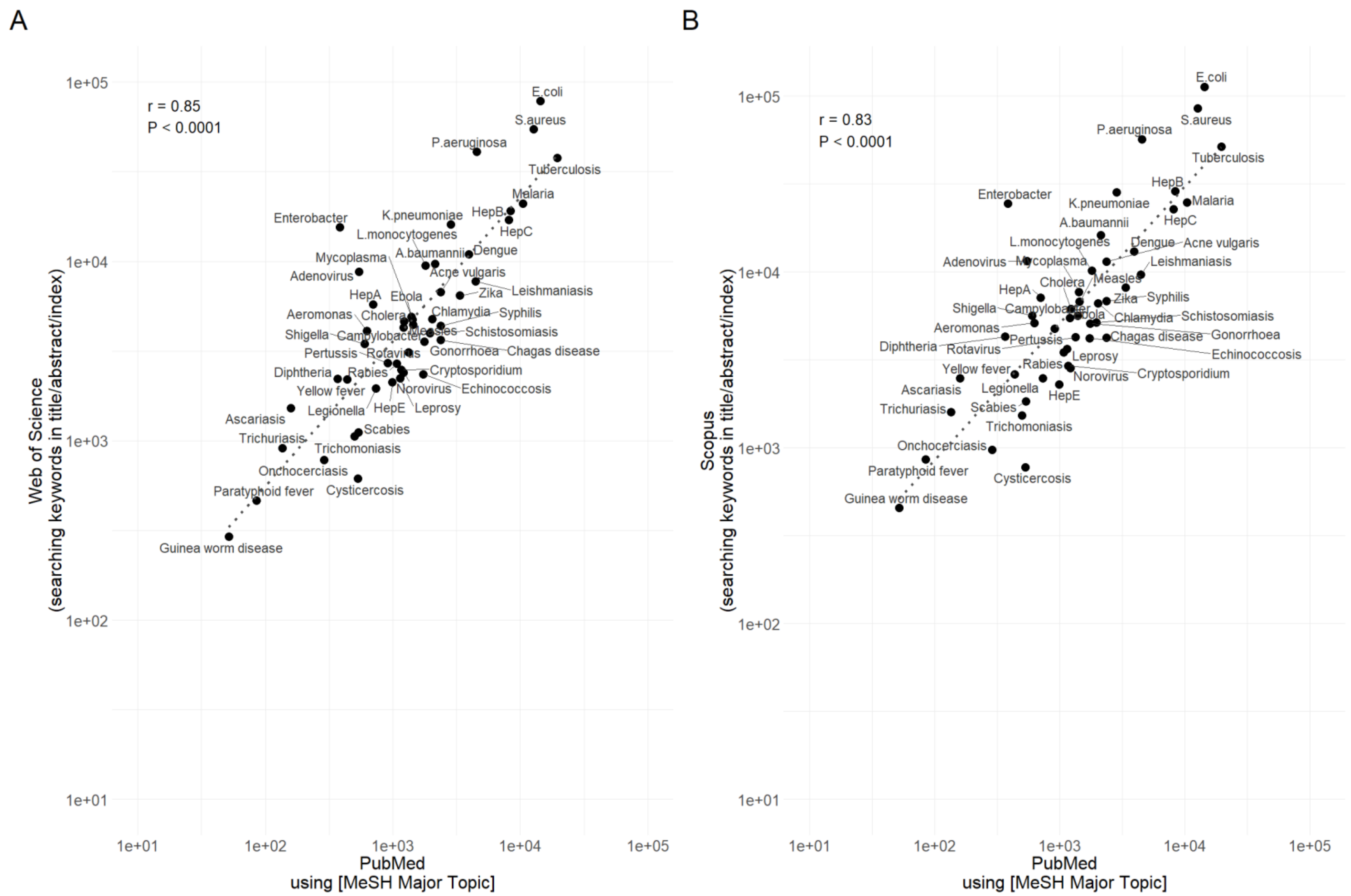


**Figure S1 Comparison of publication counts data in different databases**

The number of publications in 2020–2023 from PubMed, based on MEDLINE database, and Web of Science (panel A) or Scopus (panel B) for some diseases was plotted. P values were calculated using Pearson correlation.

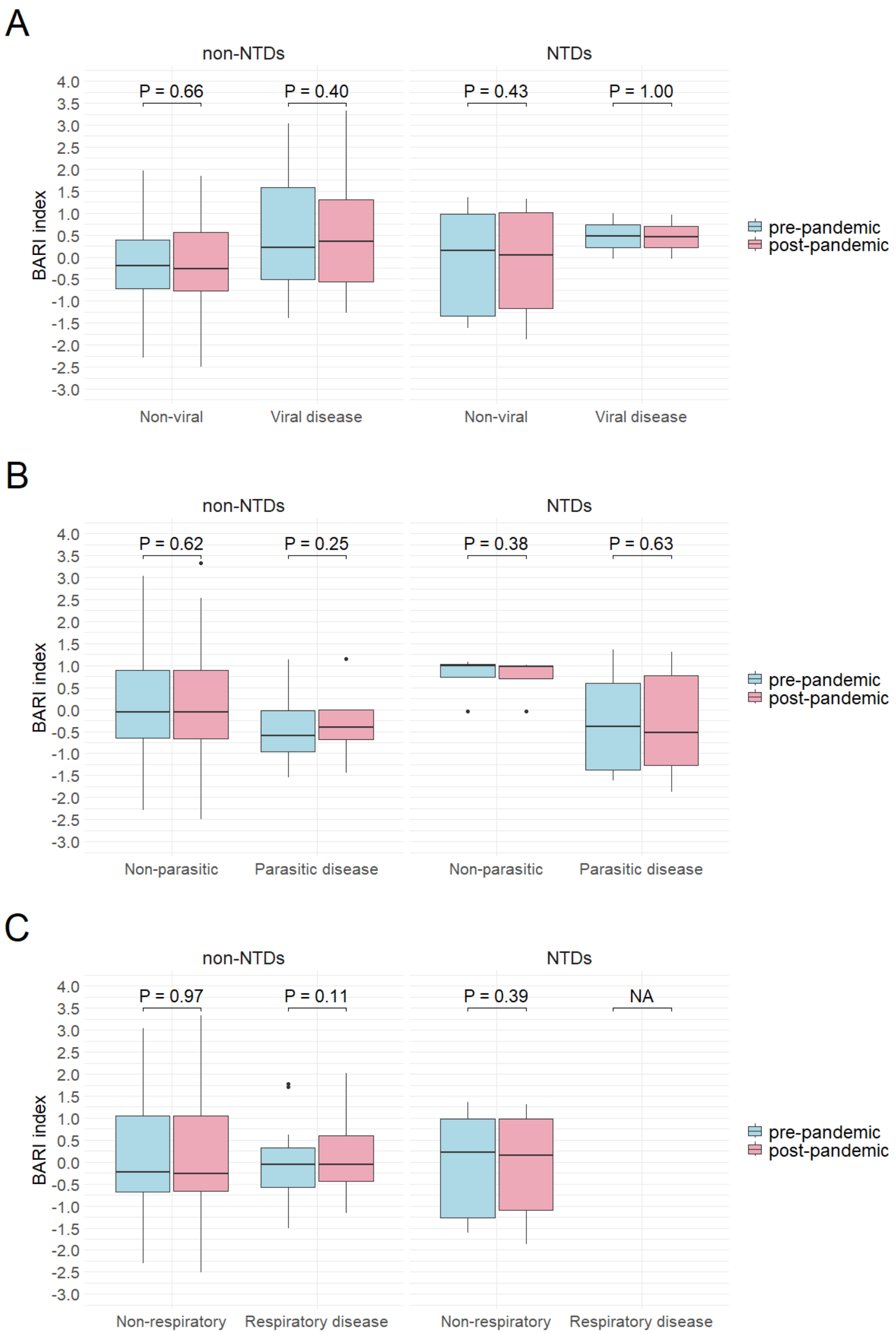


**Figure S2 BARI index comparison by disease characteristics**

The distribution of BARI index scores is shown in box plots, grouped by disease characteristics category. COVID-19 was excluded from the analyses. P values were computed using the Wilcoxon signed-rank test.

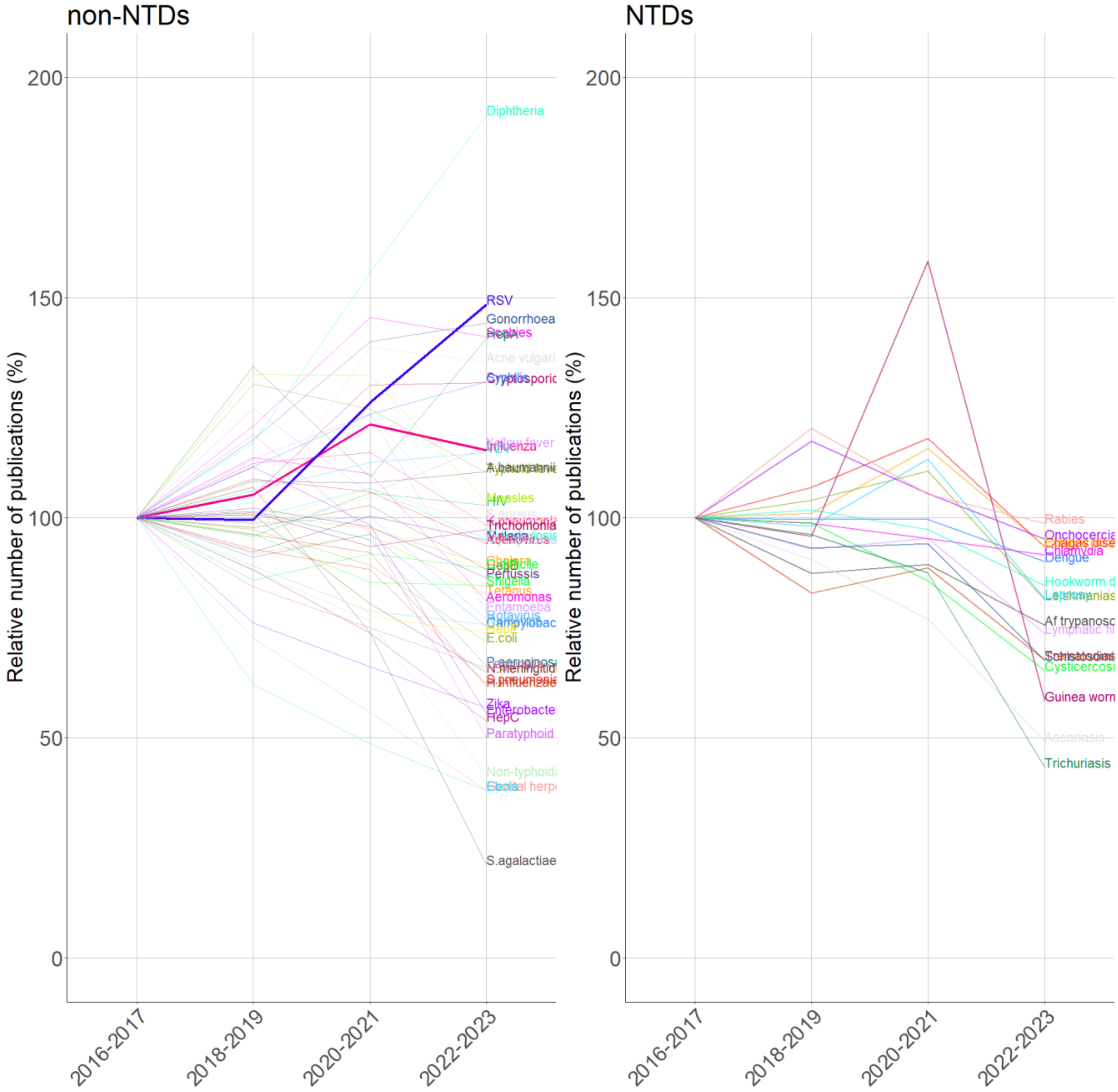


**Figure S3 Publication counts for 66 infectious diseases**

The relative number of publications for 66 infectious diseases, excluding COVID-19, in two-year window periods from 2016 to 2023, with 2016–2017 values used as a baseline, is shown for non-NTDs and NTDs. Data for influenza and RSV are indicated by thick lines.
